# Validation of differential gene expression algorithms: Application comparing fold-change estimation to hypothesis testing

**DOI:** 10.1186/1471-2105-11-63

**Published:** 2010-01-28

**Authors:** Corey M Yanofsky, David R Bickel

**Affiliations:** 1 Ottawa Institute of Systems Biology, Department of Biochemistry, Microbiology, and Immunology, University of Ottawa, Ottawa, Canada; 2 Department of Mathematics and Statistics, University of Ottawa, Ottawa, Canada

## Abstract

**Background:**

Sustained research on the problem of determining which genes are differentially expressed on the basis of microarray data has yielded a plethora of statistical algorithms, each justified by theory, simulation, or ad hoc validation and yet differing in practical results from equally justified algorithms. Recently, a concordance method that measures agreement among gene lists have been introduced to assess various aspects of differential gene expression detection. This method has the advantage of basing its assessment solely on the results of real data analyses, but as it requires examining gene lists of given sizes, it may be unstable.

**Results:**

Two methodologies for assessing predictive error are described: a cross-validation method and a posterior predictive method. As a nonparametric method of estimating prediction error from observed expression levels, cross validation provides an empirical approach to assessing algorithms for detecting differential gene expression that is fully justified for large numbers of biological replicates. Because it leverages the knowledge that only a small portion of genes are differentially expressed, the posterior predictive method is expected to provide more reliable estimates of algorithm performance, allaying concerns about limited biological replication. In practice, the posterior predictive method can assess when its approximations are valid and when they are inaccurate. Under conditions in which its approximations are valid, it corroborates the results of cross validation. Both comparison methodologies are applicable to both single-channel and dual-channel microarrays. For the data sets considered, estimating prediction error by cross validation demonstrates that empirical Bayes methods based on hierarchical models tend to outperform algorithms based on selecting genes by their fold changes or by non-hierarchical model-selection criteria. (The latter two approaches have comparable performance.) The posterior predictive assessment corroborates these findings.

**Conclusions:**

Algorithms for detecting differential gene expression may be compared by estimating each algorithm's error in predicting expression ratios, whether such ratios are defined across microarray channels or between two independent groups.

According to two distinct estimators of prediction error, algorithms using hierarchical models outperform the other algorithms of the study. The fact that fold-change shrinkage performed as well as conventional model selection criteria calls for investigating algorithms that combine the strengths of significance testing and fold-change estimation.

## Background

Continual invention of new microarray data analysis algorithms for the identification of which genes express differently across two groups calls for objectively comparing the performance of existing algorithms [1]. While there have been thorough empirical comparisons between supervised learning methods of classifying microarrays [2], comparisons between methods of detecting differential gene expression have tended to depend heavily on either theory or simulation and thus on strong underlying assumptions [3,4]. More empirical alternatives include the use of biologically-derived prior information regarding which experiments are more likely to contain differentially expressed genes [5] and the use of spike-in data sets [4,6,7]. The latter can represent equivalently expressed genes better than simulations, but the artificial spike-in levels do not necessarily correspond to levels of differentially expression across conditions of biological interest. 

An early report of the MicroArray Quality Control (MAQC) project [8] may mark a turning point in the methodology of comparing of statistical methods designed to identify differential gene expression on the basis of microarray observations. The critical advantage of this "concordance" (percentage of overlapping genes) method is its validation entirely on the basis of the microarray data without resorting to spiking in known quantities of analytes or to prior information from other types of experiments; it is thus applicable to any microarray data set with sufficient replication. Validation by non-microarray information such as RT-PCR measurements of gene expression or public pathway/functional information on genes does have great value in overcoming shortcomings in microarray platforms [9]. For that very reason, however, such validation has markedly less value in judging the performance of statistical methods of detecting differential gene expression. For example, the inability of RT-PCR to validate a microarray prediction of differential gene expression might indicate a problem with the statistical assumptions used to make the prediction, but it may instead refect a problem with cross hybridization due to the microarray platform. Participants in the MAQC project avoided such confounding between microarray platform effects and statistical method effects by quantifying the degree of overlap between gene lists produced by an algorithm on the basis of two independent data sets [8]. Although a significant step forward, this way of comparing algorithms, like that of [10], requires examining gene lists of given sizes, which is why Chen *et al*. [11] consider the concordance to be too unstable for use as an algorithm performance criterion.

Without depending on arbitrarily selected numbers of genes, the platform-algorithm confounding may be overcome by cross validation, which instead uses a test set of microarrays to validate predictions made on the basis of a separate training set of microarrays, while maintaining the empirical nature of the concordance method. Like concordance, cross validation does not incorporate knowledge that only a small portion of genes are differentially expressed. Encoding this information when appropriate into a hierarchical model enables a more reliable assessment of the performance of differential expression detection algorithms if only a few biological replicates are available. These methods are explained in Section 2 and illustrated in Section 3; implications are discussed in Section 4.

## Methods

If a gene is known to be differentially expressed at a certain level on average, then that level would predict future measurements of gene expression better than would making such predictions on the assumption that there is on average no differential expression. Likewise, if a gene is known to be equivalently expressed, then using an expression level of 0 or an expression ratio of 1 would predict future measurements better than making such predictions on the assumption that there is some differential expression. Thus, a method of selecting genes as differentially expressed may be judged by estimating its ability to predict future measurements of gene expression. This estimation may be carried out by a process of *cross validation: *the microarrays are divided between a *training set *used to determine which genes the method considers differentially expressed and a *test set *used to estimate how well such results would agree with future measurements.

The strategy of assessing gene selection algorithms by estimated prediction error may be more precisely specified in mathematical notation. Let *x*_*i*,*j *_denote the logarithm of the measured expression intensity or ratio of intensities of the *i*th of *m *genes in the *j*th of *n *biological replicates of the control or reference group; each value of *x*_*i*,*j *_may represent an average over technically replicated microarrays; **x**_*i *_= (*x*_*i*,1_,*x*_*i*,2_, ..., *x*_*i*,*n*_); **x **= (**x**_1_, **x**_2_, ..., **x**_*m*_)^*T*^. Likewise,  denotes the logarithm of the measured expression intensity or ratio of intensities of the *i*th gene in the *j*th of *n' *biological replicates of the treatment or perturbation group; . The observations *x*_*i*,*j *_and  are realizations of the random variables *X*_*i *_and , respectively. The *i*th gene is called *equivalently expressed *if E( - *X*_*i*_) = 0 or *differentially expressed *if E( - *X*_*i*_) ≠ 0. In hypothesis testing parlance, the null hypothesis associated with the *i*th gene is *H*_*i*_: E( - *X*_*i*_) = 0.

### Gene selection algorithms

A gene selection algorithm *α *returns *π*_*α *_*(H*_*i*_| **x**', **x**), an estimate of the posterior probability that the *i*th gene is equivalently expressed; it follows that 1 - *π*_*α *_(*H*_*i*_| **x**', **x**) is the algorithm's probability that the gene is differentially expressed across the perturbation and reference groups. Many algorithms [12-21] give *π*_*α *_(*H*_*i*_| **x**', **x**) directly as a local false discovery rate estimate [22,23], whereas traditional false discovery rate estimates and other non-Bayesian algorithms in effect assign *π*_*α *_(*H*_*i*_| **x**', **x**) a value of either 0 or 1, depending on whether or not a gene is considered differentially expressed at a given threshold. For example, the practice of considering a gene differentially expressed if exp , its estimated *fold change*, is at least *φ *may be expressed as(1)

with *φ *> 0, , and . The discontinuity can be removed by introducing smooth functions on an *ad hoc *basis; here we use(2)

as an example of such a smooth function. The trivial algorithms(3a)(3b)

which completely ignore the data, will serve as informative points of reference.

Some of the empirical Bayes algorithms implemented in two R packages [24] are considered here [25-27]. From calculations based on a moderated (regularized) t-statistic that are performed by the R package *limma *[25], one may readily obtain *p*_*i *_(), a one-sided p-value of the *i*th null hypothesis; **p **() = (*p*_1 _(), *p*_2 _(), ..., *p*_*m *_()). Given the moderated t-statistics and *π *(*H*_0_), the proportion of genes expected to be equivalently expressed, limma also computes log*ω*_*i *_(*π *(*H*_0_)), the estimated logarithm of the posterior odds that gene *i *is differentially expressed rather than equivalently expressed, from which the local false discovery rate may be readily obtained as (1 +*ω*_*i *_(*π *(*H*_0_)))^-1 ^. Since, for use with the log-odds, the author of the algorithm does not recommend computing *π *(*H*_0_) using limma's *convest *function (Gordon Smyth, personal communication, 27 Oct. 2007), we instead iterated the log-odds function until convergence by adapting a method [28] originally proposed for another empirical Bayes algorithm [29]:

1. Let *π*_1 _(*H*_0_) = 90% and initialize *k *to 1.

2. Increment *k *by 1.

3. Let .

4. Repeat Steps 2-3 until the absolute value of the proportion difference is sufficiently small, i.e., |*π*_*k *_(*H*_0_) - *π*_*k*-1 _(*H*_0_)| < 1/1000, or until the sign of the proportion difference changes, i.e.,

(*π*_*k *_(*H*_0_) - *π*_*k*-1 _(*H*_0_)) (*π*_*k*-1 _(*H*_0_) - *π*_*k*-2 _(*H*_0_)) < 0. The number of iterations performed until such convergence is denoted by *K*.

5. Let *π *(*H*_0_) = *π*_*K *_(*H*_0_).

Based on that value of *π *(*H*_0_), the estimated probability of equivalent expression is derived by solving for it in the definition of the odds of differential expression (i.e., the ratio of the probability of differential expression to the probability of equivalent expression), yielding(4)

Also using standard distributions of test statistics under the null hypothesis, the R package *locfdr *[26] maps **p**, a vector of single-tailed p-values for all genes, to estimates of a local false discovery rate (FDR), *π*_locfdr _(*H*_*i*_, **p**| **x**', **x**). The use of moderated t-statistics is incorporated by(5)

More commonly, **p **(*t*), a vector of standard (1- or 2-sample) t-test p-values, which also assume the normality of  - *X*_*i*_, or **p **(*w*), a vector of (signed-rank or rank-sum) Wilcoxon test p-values, which do not assume normality, yield local false discovery rate estimates(6a)(6b)

Alternatively, the *locfdr *package can employ an empirical maximum-likelihood estimate of the null distribution [27] for computation of the local-false-discovery-rate estimate *π*_emp.null _(*H*_*i*_, **p**|**x**',**x**):(7a)(7b)

Whereas the empirical Bayes algorithms provide approximations to a posterior probability of a hierarchical Bayesian class of models, we included comparisons to the posterior probability *π*_Bayes factor _(*H*_*i*_| **x**', **x**) under a non-hierarchical set of models. The data densities under the non-hierarchical models are based on the same assumptions as those of standard linear regression: unconstrained data means under the alternative hypothesis (differential expression) and, for each gene, normal IID noise and equal variance within each group in the unpaired case. Let  represent the hypothesis of differential expression (in contrast to *H_i_*, which was defined as the hypothesis of equivalent expression). The posterior odds of differential expression under these models are(8)

where *P *(d**x'**, *d****x***| *h*) is the prior predictive density or integrated likelihood under hypothesis *h*. The left-hand side of equation (8) is the posterior odds of equivalent expression to differential expression; on the right-hand side, the first factor is the prior odds of equivalent expression to differential expression, and the second factor is known as the *Bayes factor*. Since we take *P *(*H*_*i*_) = *P*() = 1/2, our posterior odds is equal to the Bayes factor; thus putting equal prior mass on each hypothesis does not share the conservatism of the above empirical Bayes algorithms. Additional file 1 gives the analytical derivation of the resulting posterior probability, which may be expressed in terms of some additional notation. Define(9)

if *n *= *n' *and  is paired with *x*_*i*,*j*_, or(10)

if  and *X*_*i *_are independent. Then the posterior probability is given by(11)(12)

We also applied two "information criteria" used in model selection to estimate the posterior probability; the information criteria were applied to the same linear regression framework used in the above Bayes factor computation. In model selection terminology, each criterion selects either model *H*_*i *_or model  (that is, equivalent expression or differential expression, respectively) for the *i*th gene, but we instead averaged the estimates corresponding to the two models for each gene as follows. We first applied the Bayesian Information Criterion (BIC) [30]. Up to a factor of - 1/2 and a constant term, the BIC approximates the logarithm of the prior predictive probability density given a statistical model and a sufficiently diffuse proper prior distribution under the given model without requiring specification of such a prior. With a prior mass on each model considered, the BIC leads to an approximation of a posterior probability that is less conservative than that of the above Bayes factor.

The general formula for the BIC under a model with normal errors is(13)

where *N *is the number of data points and *k *is the number of parameters in the model. For paired data, *N *= *n; *under *H*_*i *_the only parameter is the data variance, giving *k = *1, while under  the model includes both the data mean and data variance, giving *k = *2. Therefore the BIC for each hypothesis is(14)(15)

with *SSR*_*h *_as defined in (9).

For independent data, *N *= *n+ n'; *under *H*_*i *_the model includes a single mean log-expression level and the data variance, giving *k = *2, while under  the model includes two distinct mean log-expression levels (one for the treatment group and one for the control group) and the data variance, giving *k = *3. Therefore the BIC for each hypothesis is(16)(17)

with *SSR*_*h *_as defined in (10). Since we again use *P *(*H*_*i*_) = *P *(), the BIC approximation of the posterior odds (*ω*_*i*_,_BIC_) is equal to its approximation of Bayes factors corresponding to a wide class of priors on the model parameters. Transformed from the logarithmic scale to the probability scale [31], the result is an equation of the same form as (11),(18)(19)

The second information criterion we assessed was the Akaike Information Criterion corrected for small samples (AIC_c_). While - AIC_*c*_/2 plus a constant term is in general only an approximately unbiased estimator of the expected Kullback-Leibler distance between the model/hypothesis and the unknown true data generating distribution [32], it is exactly unbiased for linear regression models with normal errors [33], a class that includes the present non-hierarchical models. Under the name of *Akaike weights*, it and other AlC-like criteria have been used to generate predictions that take model uncertainty into account in a manner exactly analogous to Bayesian model averaging [32], giving rise to an equation of the same form as(18).

The general formula for the AIC_c _under a model with normal errors is(20)

The particular values of *N *and *k *for paired and independent data under  and *H*_*i *_are the same as those given above for the BIC. For paired data, the AIC_c _values of the hypotheses or models are(21)(22)

with *SSR*_*h *_as defined in (9); for independent data, the AIC_c _values are(23)(24)

with *SSR*_*h *_as defined in (10). Transforming from the logarithmic scale yields the effective probability(25)

Where

is the ratio of Akaike weights.

These algorithms were chosen as representatives of various classes of possible approaches. Whereas the fold-change-dependent algorithms represent algorithms that take no account of the data variance, the information criterion algorithms and the non-hierarchical Bayesian algorithm represent algorithms that do take data variance into account but do not share information across genes. The local FDR algorithms based on classical p-values share information across genes for the purpose of determining false discovery rates, thus accounting for multiple comparisons, but do not share information for estimating data variance. Algorithms employing the moderated t-statistic share information across genes to account for multiple comparisons and also to estimate data variance.

### Methods of assessing gene selection algorithms

Each of the next subsections describes a different method of quantifying the performance of gene selection algorithms. The first, cross validation, has the advantage that it is an unbiased estimator of squared prediction error (defined below) without assuming any parametric model. The second, the computation of posterior predictive loss, takes advantage of the knowledge that gene expression is approximately lognormal and that relatively few genes will have substantial differential expression, the vast majority being equivalently expressed for all practical purposes. The two methods will differ in results; if nearly all genes have only negligible differential expression, the latter is deemed more reliable except in the case of extensive biological replication since the former achieves low bias by admitting a high variance of performance estimates.

#### Cross validation

Algorithm α's best prediction of future values of  - *X*_*i *_is the posterior expected degree of expression,(26)

The term  is the best estimator of the degree of expression conditional on definite knowledge that gene *i *is differentially expressed; it is multiplied by (1 - *π*_*α *_(*H*_*i*_|**x**', **x**)), the posterior probability of differential expression. (The other product in the posterior expectation corresponds to equivalent expression, and is therefore identically zero.) The posterior expected degree of expression has been compared to a method of correcting estimates for gene selection bias [34]. For a new observation of gene *i*, the squared prediction error is,(27)

The squared prediction error does not directly target the question of which genes are differentially expressed; instead, it addresses the question of what the value of the next observation will be. However, good performance of one algorithm relative to another on either of these questions implies good performance on the other, as can be seen by considering that in general the mean squared prediction error is the sum of an algorithm's squared predictive bias and the data variance. The squared predictive bias term summarizes the ability of an algorithm to correctly distinguish differentially expressed genes from equivalently expressed genes. It is more fexible than the 0/1 loss in that it penalizes algorithms not just for being wrong, but for how wrong they are. The data variance sets the scale for "wrongness", in that for one algorithm to appear significantly worse than another, its squared predictive bias must dominate the data variance term.

Under the "all nulls false" reference algorithm, the best prediction of future values of  - *X*_*i *_for all genes is the maximum likelihood estimator . Other algorithms make gains over this reference by correctly assigning equivalently expressed genes, thereby avoiding the contribution of the variance of the MLE to the squared prediction error. Under the "all nulls true" reference algorithm, the best prediction of future values of  - *X*_*i *_for all genes is 0. Other algorithms make gains over this reference by correctly assigning differentially expressed genes, thereby avoiding the contribution of the squared bias (that is, [E ( - *X*_*i*_)]^2^) to the squared prediction error. 

The squared prediction error criterion therefore quantifies the relative costs of false positives and false negatives in terms of the bias-variance trade-off. To estimate the squared prediction error, we used leave-one-out cross validation,(28)

if *n *= *n' *and  is paired with *x*_*i*,*j *_or(29)

if  and *X*_*i *_are independent, where (-*j*) means the *j*th replicate is omitted:(30a)(30b)

, and .

For example, suppose that  is paired with *x*_*i*,,*j *_and the data for gene *i *were  = (0,1,1) and *x*_*i *_= (2,0,-2). For *j *from 1 to 3,  = (2, 0.5,-0.5), and using the fold change shrinkage calculation of equation 2, 1 - *π*_*α *_(*H*_*i*_| , **x**_(-*j*)_) = (0.95, 0.78,0.39). (Note that the FDR estimation algorithms require all the other genes' data to calculate *π*_*α *_(*H*_*i*_|, **x**_(-*j*)_).) The individual terms in the sum in equation 28 are (-2 - 0.95 - 2)^2^, (1 - 0.78 - 0.5)^2^, and (3 - 0.39 - (-0.5))^2^, and their mean is 8.6. If the given data were independent instead of paired, the calculation would involve each of the 9 subsets obtained by leaving out one perturbation data point and one control data point. 

We considered measuring error relative to always predicting that  - *X*_*i *_= 0 on a gene-wise basis using the ratio(31)

with two measures of central tendency,(32)(33)

(The half-sample mode (HSM) [35] is a fast, robust estimator of the mode that is suitable as for asymmetric distributions. It is implemented as the *hsm *function in the *modeest *package of R.) We also considered an absolute error criterion,(34)

this measure is relative to a base model such as the "all nulls true" model or the "all nulls false" model because we expect only the relative performances of the estimators to be meaningful. We found that the relative error mean essentially reproduced the absolute error relative to the "all nulls true" model, and the relative error mode often evaluated estimators as not practically different from the "all nulls true" benchmark. Therefore, we show only the results for the absolute error measure.

The use of cross-validation for estimation of classification error, appropriate for the problem of categorizing samples or microarrays given known classifications for use in the training and test sets, differs from the use cross-validation for estimation of squared prediction error, appropriate for the distinct problem of determining which genes are differentially expressed without knowledge of which genes are differentially expressed for use in the training and test sets. Jeffery *et al*. [36] used a cross-validation approach to estimate the predictive error of a variety of gene selection algorithms, but with microarray classification error rather than equations (32)-(34) as the performance criterion. Such classification error depends not only on the gene selection algorithm, but also on the particular classifier for which that algorithm selects features. Since our interest lies strictly in identifying differentially expressed genes, our methods instead quantify performance in terms of predicting new measurements. We have also addressed the problem using estimation error in place of prediction error [37].

#### Posterior predictive expected squared error

The local FDR shrinkage algorithm can be used to define an estimator's posterior predictive expected squared error. In general, the posterior predictive expected squared error is(35)

where  and *X*_*i*,*new *_are random variables for new observations,  is algorithm *α*'s point prediction for  - *X*_*i*,*new*_, and E_posterior _and var_posterior _are the expectation and variance with respect to the posterior distribution. The effective posterior distribution that leads to estimators of the form (26) is

which has variance,

We use the local FDR estimator with t-statistics and theoretical null distribution as our gold standard model for the computation of *π*_*α *_(*H*_*i*_|**x'**,**x**); this model will be accurate under the reasonable assumption that few genes are differentially expressed at appreciable levels.

To fully express the posterior predictive loss, we must define the posterior predictive distribution for  - ***X***_*new *_under both the null and alternative hypotheses for both paired and non-paired data. Conditional on each hypothesis, we use improper prior distributions for convenience. Strictly speaking, this is inconsistent with our choice of *π*_*α *_(*H*_*i*_| **x'**, **x**), an empirical Bayes approximation to a posterior probability; under a full Bayesian analysis, posterior probabilities of hypotheses can only be computed under proper priors for the parameters conditional on each hypothesis, as in the Bayes factor algorithm of equation (11). Our choice of *π*_*α *_(*H*_*i*_| **x'**,**x**) enables sharing information across genes to give a sensible empirical Bayes posterior probability for the hypotheses but otherwise relies on the same assumptions as our conditional prior distributions.

For paired data under the null hypotheses,  - ***X***_*new *_has a normal sampling distribution with zero mean and sampling variance estimated from the data. Under the usual improper prior for the sampling variance (that is, *π*_prior _(*σ*^2^)∝ *σ*^-2^), the posterior distribution for the sampling variance is a scaled-inverse-*χ*^2 ^distribution with degrees of freedom *n *and scale . The posterior predictive density is the expectation of the sampling density with respect to the posterior distribution of the sampling variance,

where N (·|·,·) is the normal distribution parameterized in terms of mean and variance, and *t*_*v *_(· |*c*,*s*^2^) is a shifted, scaled version of the *t *distribution with *v *degrees of freedom, center c, and scale factor *s*. (That is, if *Y *is distributed as *t*_*v *_(·|*c*, *s*^2^), then (*Y *- *c*)*/s *is distributed as the usual *t*_*v *_distribution.)

For paired data under the alternative hypothesis,  - *X*_*new *_has a normal sampling distribution with both mean and sampling variance estimated from the data. It can be shown that under the usual improper joint prior for mean *μ *(*μ *= E(*X' *- *X*)) and the sampling variance (that is, *π*_prior _(*μ, σ*^2^) ∝ *σ*^-2^), the posterior predictive distribution for  - *X*_*new *_is,

where , i.e., *s*^2 ^is the usual unbiased variance estimator.

For non-paired data under the null hypothesis, if the treatment and control data are modeled as having distinct sampling variances (consistent with the assumptions used to specify *π*_α _(*H*_*i*_| **x'**, **x**)) then the posterior predictive distribution is

where (*σ*')^2 ^and *σ*^2 ^are the sampling variance for treatment and control data respectively. This integral is intractable because *π*_posterior _(*σ*^2^, (*σ*')^2^)has a non-standard form (see Additional file 1). We estimated it by drawing samples from *π*_posterior _(*σ*^2^, (*σ*')^2^) using Markov chain Monte Carlo (MCMC) [38] and then calculating the MCMC average,

where the subscript *k *indicates the *k*^th ^MCMC draw of parameter values (after suitable burn-in) and *K *is the total number of draws. In the present case, the MCMC algorithm we use is an inherently multi-chain procedure; we used 10 chains. We used a burn-in of 20 samples per chain, followed by 100 samples per chain, for a total of *K = *1000 samples. For each gene in a randomly chosen subset of genes from the complete data set, a contour plot of the posterior density was superimposed on a scatter plot of the MCMC draws of parameter values. The scatter plots visually conformed to the contours of the posterior densities, verifying that the MCMC draws of parameter values provided a good approximation to the posterior distributions.

For non-paired data under the alternative hypothesis,  and *X*_*new *_each have a normal sampling distribution with both mean and sampling variance estimated from the data. It can be shown that under the usual improper joint prior for the individual means and sampling variances, the posterior predictive distributions for  and *X*_*new *_are

and therefore

To summarize gene-wise posterior predictive expected squared error over all genes in a data set, we considered quantities analogous to the relative errors and absolute errors of equations (32)-(34), with gene-wise posterior predictive expected squared errors replacing cross-validation-derived prediction errors. Again, we found that the relative error mean essentially replicated the results of the absolute error relative to the "all nulls true" benchmark; relative error mode evaluated the performance of all estimators as identical to the "all nulls true" benchmark. Therefore, we show only the results for the absolute error measure for posterior predictive expected squared error.

## Results

To illustrate the proposed methods of quantifying the performance of gene selection algorithms, we applied them to two example data sets, one relevant to agriculture and the other to medicine. Since this study is limited to the evaluation of high-level algorithms of detecting differentially expression, we did not consider multiple pre-processing schemes. The agricultural data sets were processed as described in [39]; the medical data sets were pre-processed according to the specifications of the chip manufacturer [8].

### Agricultural data

Dual-channel microarrays were used to measure in tomatoes the expression ratios (mutant/wild type) of *m *= 13,440 genes at the breaker stage of ripening and at 3 and 10 days thereafter [39]. Each of the later two stages has six biological replicates (*n *= 6), but one of the biological replicates is missing at the breaker stage of ripening (*n *= 5). The next subsection compares algorithms of determining which genes are differentially expressed between mutant and wild type at each point in time, whereas Subsection 3.2 uses the same data to instead compare algorithms of determining which genes are differentially expressed between one point in time and another point in time.

#### Pairing across microarray channels

In order to determine the genes for which expected values of logarithms of mutant-to-wild-type ratios differ from 0, let  be the expression level of the mutant sample with mRNA hybridized to the same microarray as that of a wild type sample with expression level *x*_*i*,*j *_at 0, 3, or 10 days after the breaker stage. Then  - *x*_*i*,*j *_is the logarithm of the observed ratio for the *i*th gene and *j*th microarray. Due to this dependence structure, paired (1-sample) t-tests and Wilcoxon signed-rank tests were used to obtain p-values, and equation (34) was used to estimate prediction error. We measured absolute error relative to the local FDR using the t-statistic and the theoretical null (labelled "t stat. with locfdr") because this model had the best or near-best performance in seven out of the nine data sets considered in this paper. Thus, its use as the base model facilitated the plotting of multiple data sets in a single figure; this same model was used as the base model for all subsequent figure. The estimated prediction errors for all algorithms mentioned above are displayed as Figure 

**Figure 1:**
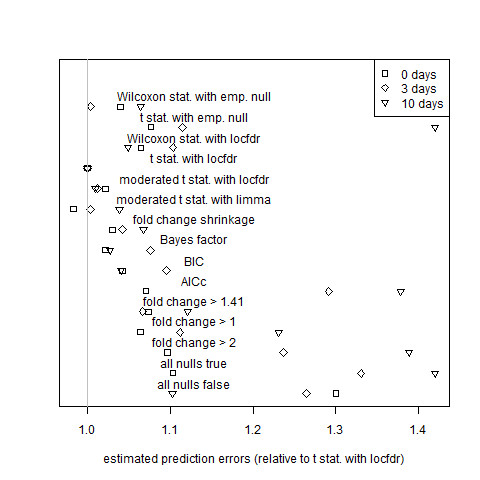
**Assessment of estimator performance by cross validation for the paired tomato data sets**. Average estimated prediction error, defined by equation (34) and based on cross validation, at the breaker stage of ripening (squares), 3 days after ripening (diamonds) and 10 days after ripening (triangles). The values of *α *displayed correspond to the gene selection algorithms of equations (1)-(7).

**Figure 2:**
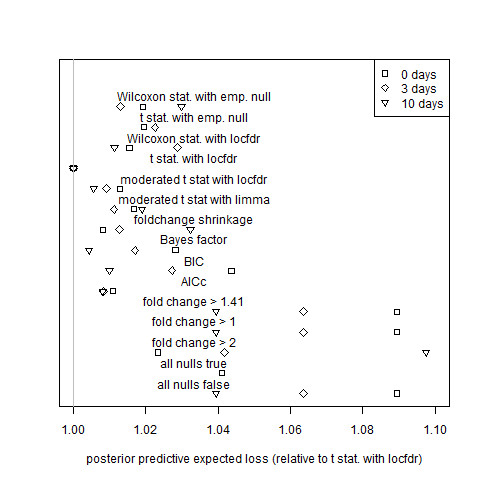
**Assessment of estimator performance by posterior predictive expected squared error for the paired tomato data sets**. Total posterior predictive squared error (defined by equation (34)) relative to that of the gold standard model (the local-FDR mean expression estimator calculated using t-statistics and the theoretical null, labeled "t stat. with locfdr" in the figure) for 0, 3, and 10 day tomato data sets. Algorithm definitions are the same as those of Figure 1. Results for the 3 day and 10 day tomato data sets with the "all nulls true" estimator are greater than 1.1 and are not plotted.

#### Two independent groups

**Figure 3:**
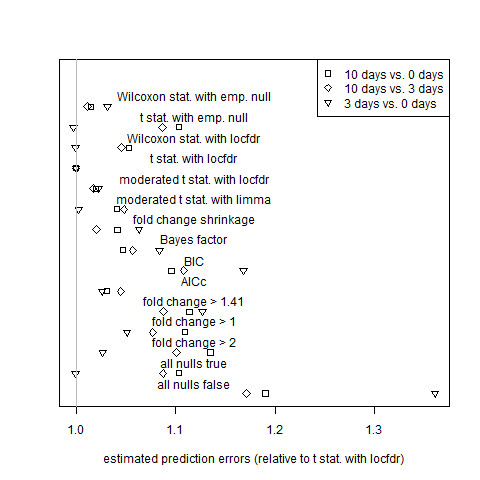
**Assessment of estimator performance by cross validation for the non-paired tomato data sets**. Average estimated prediction error for the comparing expression at 10 days to 0 days, 10 days to 3 days, and 3 days to 0 days after the breaker stage of ripening. Error and algorithm definitions are the same as those of Figure 1.

**Figure 4:**
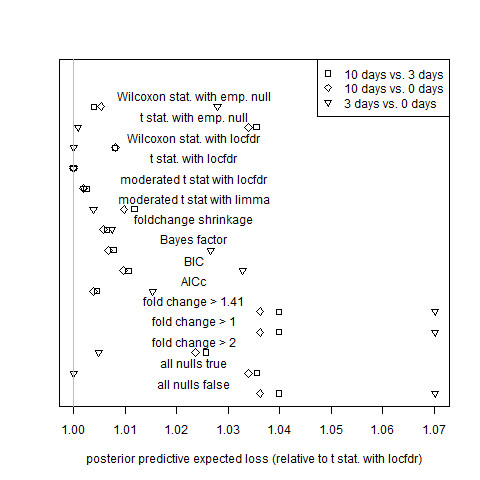
**Assessment of estimator performance by posterior predictive expected squared error for the non-paired tomato data sets**. Total posterior predictive squared error relative to that of the gold standard model (the local-FDR mean expression estimator calculated using t-statistics and the theoretical null, labeled "t stat. with locfdr" in the figure) for 0 days vs. 3 days, 0 days vs. 10 days, and 3 days vs. 10 days tomato data sets. Error definitions are the same as those of Figure 2. Algorithm definitions are the same as those of Figure 1.

In order to determine which genes differ in mutant-to-wild-type ratios between different periods of time after the breaker stage, let  and *x*_*i*,*j *_denote the logarithms of ratios observed at two different points in time for gene *i *and for microarrays *j' *and *j*. Since the measurement errors of observations made at one time point are independent of those made at the other time point, 2-sample t-tests and Wilcoxon rank-sum tests were used to obtain p-values, and equation (34) was used to average estimated prediction error (Figure 3). Figure 4 shows, for each non-paired tomato data set, the total posterior predictive expected squared error (equation (35)) for each estimator relative to that of the gold standard model.

### Biomedical data

**Figure 5:**
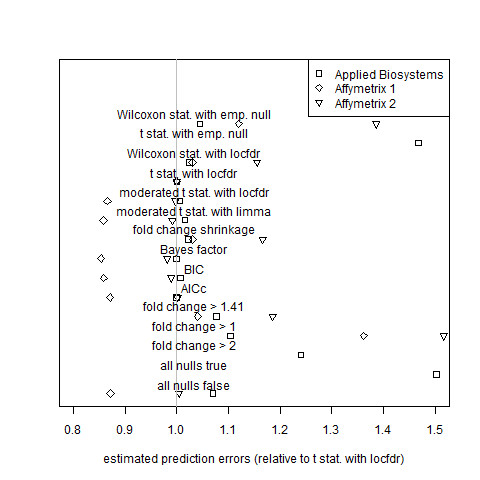
**Assessment of estimator performance by cross validation for the MAQC data sets**. Average estimated prediction error for the Applied Biosystems, Affymetrix 1, and Affymetrix 2 data sets of the rat toxicogenomics subset of the MAQC study. Error and algorithm definitions are the same as those of Figure 1. Results for both Affymetrix data sets with the "fold change > 4" hard threshold estimator, the "all nulls true" estimator, and the local FDR estimator based on *t *statistics and the empirical null are greater than 1.7 and are not plotted.

**Figure 6:**
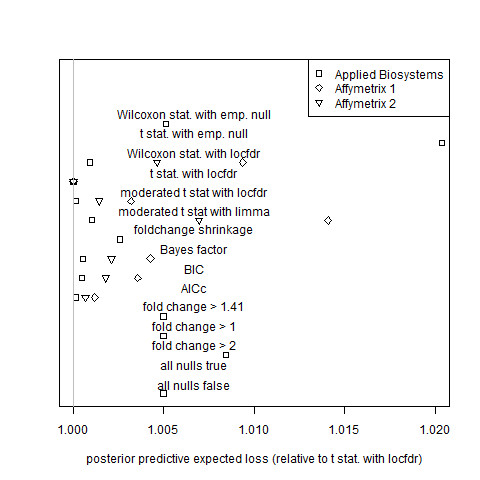
**Assessment of estimator performance by posterior predictive expected squared error for the MAQC data sets**. Total posterior predictive squared error relative to that of the gold standard model (the local-FDR mean expression estimator calculated using t-statistics and the theoretical null, labeled "t stat. with locfdr" in the figure) for Applied Biosystems, Affymetrix 1, and Affymetrix 2 data sets of the rat toxicogenomics subset of the MAQC study. Error definitions are the same as those of Figure 2. Algorithm definitions are the same as those of Figure 1. Results for the Applied Biosystems data set with the "all nulls true" estimator and for the both Affymetrix data sets with the local FDR estimator with empirical null (based on both Wilcoxon statistics and t statistics), the fold change shrinkage estimator, all fold change hard threshold estimators, the "all nulls true" estimator, and the "all nulls false" estimator are greater than 1.023 and are not plotted.

MAQC researchers [8] measured gene expression responses to a rat liver treatment on four different platforms: Applied Biosystems, Affymetrix, Agilent, and GE Healthcare. Each data set has six treatment biological replicates and six control biological replicates. As in Subsection 3.1.2, observations in the treatment group are not paired with those of the control group. The Applied Biosystems data set (*m *= 26,857 genes) and the two Affymetrix data sets (*m *= 31, 099 genes each) were used to assess gene selection criteria on the basis of prediction error (Figure 5). Figure 6 shows, for each MAQC data set, the total posterior predictive expected squared error (equation (35)) for each estimator relative to that of the gold standard model.

## Discussion

### Fold change versus testing

Fold change performs about as well as simple (non-hierarchical) model selection criteria except when it is penalized by the imposition of a hard threshold. Algorithms based on hard thresholds for fold change are outperformed by shrinkage fold-change and by all other non-trivial algorithms that are not restricted by arbitrary thresholds: Tables 1 and 2 show that hard-threshold algorithms are never ranked in the top four by either cross validation or posterior predictive expected loss. While the best local-FDR-based methods outperform shrinkage fold-change, as can be seen in Figures 1, 2, 3, 4, 5 and 6, shrinkage fold change has performance comparable to simple model selection criteria as represented by the Bayes factor, BIC, and AIC_c _methods. 

Herein we examined only algorithms that fall into one of two distinct categories:

**Table 1:** Number of tomato data sets for which each estimator ranked in the top four.

algorithm	cross validation posterior	predictive expected loss
Wilcoxon stat. with emp. null	3	2

t stat. with emp. null	1	1

Wilcoxon stat. with locfdr	1	1

t stat. with locfdr	6	6

moderated t stat. with locfdr	5	5

moderated t stat. with limma	3	1

fold change shrinkage	1	1

Bayes factor	2	1

BIC	0	0

AICc	1	5

fold change > 1.41	0	0

fold change > 1	0	0

fold change > 2	0	0

all nulls true	1	1

all nulls false	0	0

**Table 2:** Number of MAQC data sets for which each estimator ranked in the top four.

algorithm	cross validation posterior	predictive expected loss
Wilcoxon stat. with emp. null	0	0

t stat. with emp. null	0	0

Wilcoxon stat. with locfdr	0	0

t stat. with locfdr	1	3

moderated t stat. with locfdr	3	3

moderated t stat. with limma	2	0

fold change shrinkage	0	0

Bayes factor	3	0

BIC	2	3

AICc	1	3

fold change > 1.41	0	0

fold change > 1	0	0

fold change > 2	0	0

all nulls true	0	0

all nulls false	0	0

1. The shrinkage and hard-threshold fold-change algorithms are based on estimated fold change without regard for statistical significance or estimates of variance.

2. All other algorithms of the present study compute levels of significance without regard for fold change estimates. (We converted the results of these algorithms into predictions for the sole purpose of comparing the predictive performance of different algorithms.)

Since these categories represent opposite extremes, their algorithms might be outperformed by those that instead employ both fold-change information and variance/significance information. Our observation that fold change performs as well as simple model-selection criteria suggests consideration of less extreme algorithms that combine the advantages of the ones studied herein. Investigators reported that the estimation of fold-change following a non-stringent significance filter performs better than does either type of algorithm alone [8,40], and [37] have recently demonstrated that further improvement is possible by smoothly shrinking estimates of fold change according to statistical significance levels.

Adjusting fold-change estimates according to significance levels is not the only way to combine the two types of information. A complementary strategy instead adjusts significance levels according to fold-change thresholds. In fact, the seemingly inferior performance of statistical methods that do not make use of fold-change estimates has been explained in terms of a distinction between statistical and biological significance [41], which would call for the incorporation of the lowest fold change considered biologically relevant into the statistical hypotheses under consideration. Recent statistical methods designed to find genes expressed at biologically important levels include those utilizing false discovery rates [42,43], empirical and full Bayesian analyses [44-46], and the likelihood paradigm of measuring the strength of statistical evidence [47].

### Corroboration of cross validation by posterior predictive expected loss

In general, cross-validation is subject to high variance when sample sizes are small. If each of the features had independent data of finite variance, the central limit theorem would nonetheless guarantee a small variance in the overall measure of performance (34). In the present case, however, due to gene-gene interactions, the numerator and denominator of the overall measure of performance are sums of positively correlated quantities. To address this concern, we performed an additional assessment of the differential-expression-detection algorithms using posterior predictive expected squared error methodology. 

The posterior predictive expected squared error requires the choice of a particular gold standard model, a Bayesian model consisting of a family of sampling distributions refecting knowledge about the biological system and a prior distribution. Here, we based our posterior predictive expected squared error on the implicit Bayesian model approximated by the local-FDR mean expression estimator calculated using t-statistics and the theoretical null distribution. The key assumption of the model is that few genes are differentially expressed at any notable level; the model also assumes that gene expression ratios are lognormally distributed. The model accommodates unequal variances for non-paired data using conventional improper priors under each hypothesis since we have little prior information about the specific parameter values. (As such priors are arbitrary and carry their own information, a more thorough Bayesian analysis would require a study of the sensitivity of results to the choice of prior.) Naturally, the model's corresponding estimator had the lowest posterior predictive expected squared error, but provided the assumptions encoded in the model hold, the posterior predictive expected squared error will nonetheless be a good way to rank the performance of the estimators.

The fitting of the gold-standard model generated estimates for the proportions of equivalently expressed genes, allowing the verification of the assumption that most genes were equivalently expressed. For the 0 days, 3 days, and 10 days data sets, the estimated proportion of equivalently expressed genes were 0.91, 0.89, and 0.73, respectively; for the 10 days vs. 3 days, 10 days vs. 0 days, and 3 days vs. 0 days data sets, they were 0.83, 0.83, and 1.00, respectively; and for the Applied Biosystems, Affymetrix 1, and Affymetrix 2 data sets, they were 0.62, 0.59, and 0.60, respectively. The six tomato data sets have relatively high proportions, showing that these data sets more closely satisfy the assumption of a proportion close to 1. Therefore, the local-FDR-based rankings for the estimators in these data sets should be accurate. The MAQC data sets have lower proportions, indicating that the model assumption is a poor approximation. It is not surprising that the MAQC data sets have many differentially expressed genes, as they are derived from liver tissue treated with a potent toxin.

As noted before, the cross-validation performance measure ranks the gold standard model highly for the tomato data sets, that is, for the data sets that we expect good estimation from the gold standard model. Furthermore, a careful inspection of Figures 1, K2, K3 and K4 revealed that the rankings of the estimators according to the posterior predictive assessment and the cross-validation assessment largely agreed. (Some notable exceptions were the AIC_c_, which was rated highly by posterior predictive expected loss but poorly by cross-validation for the 0 days and 3 days data sets (Figures 1-2), and the moderated t-statistic with limma, which was rated highly by cross-validation but poorly by posterior predictive expected loss for the 0 days, 3 days, 10 days vs. 3 days, and 3 days vs. 0 days data sets (Figures 1, K2, K3 and K4).) In addition, the cross-validation performance measure does not rank the gold standard model as highly for the Affymetrix data sets; the gold standard model itself has determined that its assumption of a high proportion of equivalently expressed genes fails for precisely those data sets. (The Applied Biosystems data set is unusual in that its median gene variance was roughly five times larger than the median gene variances of the other data sets. As a result, there is little power to distinguish between estimators: the gold standard model estimator, the Bayes Factor estimator, and the AIC_c _estimator are essentially tied for best performance (Figure 5) These observations suggest that the cross-validation methodology was able to accurately rank estimators even though the number of biological replicates was small.

## Conclusion

The posterior predictive methodology helped to confirm that the cross-validation methodology was effective for measuring estimators' relative performances. The results support the use of local-FDR-based statistical algorithms over both conventional model-select ion criteria and over algorithms based only on fold change. In particular, the estimator based on the local FDR calculated using t-statistics and the theoretical null had the overall best performance when the proportion of equivalently expressed genes was high. As a second choice, the estimator based on the local FDR calculated using moderated t-statistics also performed quite well. Tables 1 and 2 show that it was ranked in the top four for eight data sets out of nine, including all three MAQC data sets.

The fact that fold-change shrinkage performed as well as conventional model selection criteria calls for investigating algorithms that combine the strengths of significance testing and fold-change estimation.

## Authors' contributions

CMY selected the Bayes factor algorithm, implemented the Bayes factor, BIC, and AIC_c _algorithms, and implemented the posterior predictive expected squared loss assessment. DRB conceived the study, selected the data sets, and applied the fold change and empirical Bayes algorithms. Each author made substantial contributions to writing the paper. Both authors read and approved the final manuscript.

## Supplementary Material

Additional file 1 This file contains a heuristic overview and detailed derivation of our Bayes factor approach to calculating probabilities of differential expression. Click here for file
