# Supplementary material for: Validation of differential gene expression algorithms: Application comparing fold-change estimation to hypothesis testing
Source: BMC Bioinformatics. 2010 Jan 28;11:63. doi: 10.1186/1471-2105-11-63 (PMC3224549; doi:10.1186/1471-2105-11-63)
Supplement: Additional file 1 — This file contains a heuristic overview and detailed derivation of our Bayes factor approach to calculating probabilities of differential expression. [file 1471-2105-11-63-S1.DOCX]

## Supplementary Information

Validation of differential gene expression algorithms: Application comparing fold-change estimation to hypothesis testing

Corey M. Yanofsky and David R. Bickel

## A.1. Heuristic overview of the derivation of the Bayes factor

This document contains an explicit derivation of the Bayes factor used in the main paper for both paired and unpaired data. In each case, there are two models for the data: the null model in which the gene is equivalently expressed in the two conditions, and the alternative model in which the gene is differentially expressed.

The derivation of the Bayes factor requires two components per model. The first component is the probability distribution of the data conditional on some statistical parameters; this is termed the *likelihood function*. The differential expression model will always have one extra parameter to take into account the fact that the gene’s expression level is different across conditions.

The data are always modeled as:

*observed datum* = *average data level* + *measurement error*.

Here, the average data level is an unknown parameter. Throughout, the measurement errors are assumed to be independent and identically distributed as Gaussian random variables. That is, for all *j*,

$p\left( εε\varepsilon_{j}\left| \sigma^{2} \right. \right)=\frac{1}{\sigma\sqrt{2\pi}}\exp\left[ -\frac{1}{2\sigma^{2}}{\varepsilon_{j}}^{2} \right]$,

where $\varepsilon_{j}$ is the measurement error of the *j*^th^ observation and $\sigma^{2}$ is the data variance .

The second component is the prior distribution of the model parameters, namely, the baseline expression level of the gene and the experimental variability of the data. The prior distribution summarizes everything that is known about the model parameters prior to observing the data. Since the basic expression level of the gene and the variability of the data are unknown, we use standard default priors for them.

The extra parameter in the alternative model measures the amount of differential expression. Here, we use what has been called a *unit-information* prior distribution, that is, a prior distribution that contains exactly as much information as one extra data point. The unit-information prior is weakly informative, so it will not unduly influence the results in favor of either model.

To calculate the Bayes factor, we *marginalize* the model parameters; that is, we integrate the likelihood function with respect to the prior distribution, resulting in a *prior predictive distribution*. The marginalization removes the nuisance parameters from the expression. The Bayes factor is the ratio of the prior predictive distributions under the null and alternative models.

## A.2 Derivations

## Bayes factor for paired data

Suppose *n* = *n*′ and $x_{i,j}^{'}$ is paired with $x_{i,j}.$Let

$y_{j}=x_{i,j}^{'}-x_{i,j}$. (S1)

The hypothesis of equivalent expression is

*M*_0_: *y_j_* = *ε_j_,*

and the hypothesis of differential expression is

*M*_1_: *y_j_* = *α* + *ε_j_*.

Prior distributions

For both models, we set

$p\left( \sigma^{2} \right)\propto\frac{1}{\sigma^{2}}$.

For *M*_1_, we use the unit information prior

$$p\left( \alpha\left| \sigma^{2} \right. \right)=\frac{1}{\sigma\sqrt{2\pi}}\exp\left[ -\frac{1}{2\sigma^{2}}\left( \alpha-\mu_{\alpha} \right)^{2} \right].$$

In the main text of the paper, the prior mean $\mu_{\alpha}$ is set to zero.

Null model prior predictive distribution

The prior predictive distribution of the data under *M*_0_ is

$$p\left( y | M_{0} \right)=\int_{0}^{\infty} p\left( \sigma^{2} \right)\prod_{j} p\left( y_{j}\left| \mu=0,\sigma^{2} \right. \right)d\sigma^{2},$$

$$p\left( y | M_{0} \right)=\left( 2\pi\right)^{-n/2}\int_{0}^{\infty} \left( \frac{1}{\sigma^{2}} \right)^{\frac{n}{2}+1}\exp\left( -\frac{n\bar{y^{2}}}{{2\sigma}^{2}} \right)d\sigma^{2},$$

$p\left( y\left| M_{0} \right. \right)=\left( 2\pi\right)^{-n/2}\text{}\left( \frac{n}{2} \right)\left( n\bar{y^{2}} \right)^{-n/2}$.

Alternative model prior predictive distribution

We define in advance:

$\hat{\alpha}=\frac{\left( n\bar{y}+\mu_{\alpha} \right)}{n+1}$,

$${SSR}_{1}=\left( \mu_{\alpha}-\hat{\alpha} \right)^{2}+\sum_{j} \left( y_{j}-\hat{\alpha} \right)^{2}.$$

After some algebra, we can derive

$${SSR}_{1}=n\left( \bar{y^{2}}-\left( \bar{y} \right)^{2} \right)+\frac{n}{n+1}\left( \bar{y}-\mu_{\alpha} \right)^{2}.$$

Here, *SSR*_1_ is the effective sum of squares of the residuals under *M*_1_. It is the sum of the *SSR* using the maximum likelihood estimator $\alpha_{MLE}=\bar{y}$ and a term that penalizes disagreement between the MLE and the prior mean.

The prior predictive distribution of the data under *M*_1_ is

$$p\left( y | M_{1} \right)=\int_{0}^{\infty} \int_{-\infty}^{\infty} p\left( \sigma^{2} \right)p\left( \alpha\left| \sigma^{2} \right. \right)\prod_{j} p\left( y_{j}\left| \mu=\alpha,\sigma^{2} \right. \right)d\alpha d\sigma^{2},$$

$$p\left( y | M_{1} \right)=\left( 2\pi\right)^{-\left( n+1 \right)/2}\int_{0}^{\infty} \left( \frac{1}{\sigma^{2}} \right)^{\frac{\left( n+1 \right)}{2}+1}\left( \int_{-\infty}^{\infty} \exp\left\{ -\frac{1}{2\sigma^{2}}\left[ \left( \alpha-\mu_{\alpha} \right)^{2}+\sum_{j} \left( y_{j}-\alpha\right)^{2} \right] \right\}d\alpha\right)d\sigma^{2}.$$

Isolating the part of the integrand that is a quadratic expression in *α*, we complete the square:

$$\left( \alpha-\mu_{\alpha} \right)^{2}+\sum_{j} \left( y_{j}-\alpha\right)^{2}$$

$$=\alpha^{2}-2\alpha\mu_{\alpha}+{\mu_{\alpha}}^{2}+n\alpha^{2}-2\alpha n\bar{y}+n\bar{y^{2}}$$

$$=\left( n+1 \right)\alpha^{2}-2\alpha\left( n\bar{y}+\mu_{\alpha} \right)+\left( n\bar{y^{2}}+{\mu_{\alpha}}^{2} \right)$$

$$=\left( n+1 \right)\left( \alpha-\hat{\alpha} \right)^{2}+\left( n\bar{y^{2}}-n\left( \bar{y} \right)^{2} \right)+{\mu_{\alpha}}^{2}+n\left( \bar{y} \right)^{2}-\frac{n^{2}\left( \bar{y} \right)^{2}+2n\bar{y}\mu_{\alpha}+{\mu_{\alpha}}^{2}}{n+1}$$

$$=\left( n+1 \right)\left( \alpha-\hat{\alpha} \right)^{2}+n\left( \bar{y^{2}}-\left( \bar{y} \right)^{2} \right)+\frac{n{\mu_{\alpha}}^{2}+n^{2}\left( \bar{y} \right)^{2}+{\mu_{\alpha}}^{2}+n\left( \bar{y} \right)^{2}}{n+1}-\frac{n^{2}\left( \bar{y} \right)^{2}+2n\bar{y}\mu_{á}+{\mu_{\alpha}}^{2}}{n+1}$$

$$=\left( n+1 \right)\left( \alpha-\hat{\alpha} \right)^{2}+n\left( \bar{y^{2}}-\left( \bar{y} \right)^{2} \right)+\frac{1}{n+1}\left( {n\left( \bar{y} \right)}^{2}-2n\bar{y}\mu_{\alpha}+{{n\mu}_{\alpha}}^{2} \right)$$

$$=\left( n+1 \right)\left( \alpha-\hat{\alpha} \right)^{2}+n\left( \bar{y^{2}}-\left( \bar{y} \right)^{2} \right)+\frac{n}{n+1}\left( \bar{y}-\mu_{\alpha} \right)^{2}$$

$=\left( n+1 \right)\left( \alpha-\hat{\alpha} \right)^{2}+{SSR}_{1}$.

Substituting back into the integral, we have

$p\left( y | M_{1} \right)=\left( 2\pi\right)^{-\left( n+1 \right)/2}\int_{0}^{\infty} \left( \frac{1}{\sigma^{2}} \right)^{\frac{\left( n+1 \right)}{2}+1}\exp\left( -\frac{{SSR}_{1}}{2\sigma^{2}} \right)\left( \int_{-\infty}^{\infty} \exp\left[ -\frac{n+1}{2\sigma^{2}}\left( \alpha-\hat{\alpha} \right)^{2} \right]d\alpha\right)d\sigma^{2}$,

$p\left( y | M_{1} \right)=\left( 2\pi\right)^{-n/2}\left( n+1 \right)^{{-1}/2}\int_{0}^{\infty} \left( \frac{1}{\sigma^{2}} \right)^{\frac{n}{2}+1}\exp\left( -\frac{{SSR}_{1}}{2\sigma^{2}} \right)d\sigma^{2}$,

$p\left( y\left| M_{1} \right. \right)=\left( 2\pi\right)^{-n/2}\text{}\left( \frac{n}{2} \right){\left( n+1 \right)^{{-1}/2}\left( {SSR}_{1} \right)}^{-n/2}$.

The Bayes factor is

$$BF=\frac{p\left( y\left| M_{0} \right. \right)}{p\left( y\left| M_{1} \right. \right)}=\sqrt{n+1}\left( \frac{{SSR}_{1}}{n\bar{y^{2}}} \right)^{\frac{n}{2}}. (S2)$$

Equations (S1) and (S2) together are equivalent to equations (9), (11), and (12) of the main paper.

## Bayes factor for two-sample data

Suppose that $X_{i,j}^{'}$ and $X_{i,j}$ are independent. Define

$y_{j}=x_{i,j}, j=1,\ldots, n$*,* (S3)

$y_{n+j}=x_{i,j}^{'}, j=1,\ldots, n'$ *.* (S4)

The hypothesis of equivalent expression is

*M*_0_: $y_{j}=\beta+\varepsilon_{j}, j=1,\ldots, n+n'$*,*

and the hypothesis of differential expression is

*M*_1_: $y_{j}=\beta+\varepsilon_{j}, j=1,\ldots, n$,

$y_{j}=\alpha+\varepsilon_{j}, j=n+1,\ldots, n+n'$.

Preliminaries

To fix notation, let

$$\bar{y^{2}}=\frac{1}{n+m}\sum_{j=1}^{n+n'} {y_{j}}^{2},$$

$$\bar{y}=\frac{1}{n+m}\sum_{j=1}^{n+n'} y_{j},$$

$$\bar{y_{a}}=\frac{1}{n'}\sum_{j=n+1}^{n+n'} y_{j},$$

$$\bar{y_{b}}=\frac{1}{n}\sum_{j=1}^{n} y_{j}.$$

Before beginning the derivation of the Bayes factor, we note that the maximum likelihood estimates under *M*_1_ are

$\alpha_{MLE}=\bar{y_{a}}$,

$\beta_{MLE}=\bar{y_{b}}$,

and the sum of squares of the residuals using the MLEs is

$${SSR}_{MLE}=\left( n+m \right)\bar{y^{2}}-m\left( \bar{y_{a}} \right)^{2}-n\left( \bar{y_{b}} \right)^{2}.$$

Prior distributions

For both models, we set the prior for $\left( {\beta,\sigma}^{2} \right)$ to be

$p\left( {\beta,\sigma}^{2} \right)\propto\frac{1}{\sigma^{2}}$.

For the extra parameter in *M*_1_, we use the unit information prior centered at $\alpha=\beta$,

$p\left( \alpha\left| {\beta,\sigma}^{2} \right. \right)=\frac{1}{\sigma\sqrt{2\pi}}\exp\left[ -\frac{1}{2\sigma^{2}}\left( \alpha-\beta\right)^{2} \right]$.

Null model prior predictive distribution

The prior predictive probability of the data under *M*_0_ is

$$p\left( y | M_{0} \right)=\int_{0}^{\infty} p\left( {\beta,\sigma}^{2} \right)\int_{-\infty}^{\infty} \prod_{j=1}^{n+n'} p\left( y_{j}\left| \beta,\sigma^{2} \right. \right)d\beta d\sigma^{2},$$

$$p\left( y | M_{0} \right)=\left( 2\pi\right)^{-\frac{n+n'}{2}}\int_{0}^{\infty} \left( \frac{1}{\sigma^{2}} \right)^{\frac{n+n'}{2}+1}\exp\left( -\frac{\left( n+n' \right)\left( \bar{y^{2}}-\left( \bar{y} \right)^{2} \right)}{2\sigma^{2}} \right)\left( \int_{-\infty}^{\infty} \exp\left( -\frac{n\left( \beta-\bar{y} \right)^{2}}{2\sigma^{2}} \right)d\beta\right)d\sigma^{2},$$

$$p\left( y | M_{0} \right)=\left( 2\pi\right)^{-\frac{n+n'-1}{2}}\left( n+n' \right)^{-\frac{1}{2}}\int_{0}^{\infty} \left( \frac{1}{\sigma^{2}} \right)^{\frac{n+n'-1}{2}+1}\exp\left( -\frac{\left( n+n' \right)\left( \bar{y^{2}}-\left( \bar{y} \right)^{2} \right)}{2\sigma^{2}} \right)d\sigma^{2},$$

$$p\left( y\left| M_{0} \right. \right)=\left( 2\pi\right)^{-\frac{n+n'-1}{2}}\left( n+n' \right)^{-\frac{1}{2}}\text{}\left( \frac{n+n'-1}{2} \right)\left[ \left( n+n' \right)\left( \bar{y^{2}}-\left( \bar{y} \right)^{2} \right) \right]^{-\left( n+n'-1 \right)/2}.$$

Alternative model prior predictive distribution

We define in advance:

$$E\left( \alpha| \beta,y \right)=\frac{\left( n'\bar{y_{a}}+\beta\right)}{n'+1},$$

$$\hat{\beta}=\left( \frac{\left( n+nn' \right)\bar{y_{b}}+n'\bar{y_{a}}}{n+n^{'}+nn'} \right),$$

$${SSR}_{1}={SSR}_{MLE}+\frac{nn'}{\left( n+n^{'}+nn' \right)}\left( \bar{y_{a}}-\bar{y_{b}} \right)^{2}.$$

As before, the effective sum of squares of the residuals under *M*_1_ is the sum of the *SSR* using the maximum likelihood estimators and a penalty term for disagreement between the MLEs and the prior distribution.

Before dealing with the marginal probability of the data under *M*_1_, we re-arrange the quadratic expression in *α* and *β* to ease the integrations.

$$\left( \alpha-\beta\right)^{2}+\sum_{j=1}^{n} \left( y_{j}-\beta\right)^{2}+\sum_{j=n+1}^{n+n'} \left( y_{j}-\alpha\right)^{2}$$

$$={\alpha^{2}+\beta}^{2}-2\alpha\beta+n\beta^{2}-2n\bar{y_{b}}\beta+n'\alpha^{2}-2n'\bar{y_{a}}\alpha+\left( n+n' \right)\bar{y^{2}}$$

$$=\left( n+1 \right)\beta^{2}-2n\bar{y_{b}}\beta+\left( n'+1 \right)\alpha^{2}-2\left( n'\bar{y_{a}}+\beta\right)\alpha+\left( n+n' \right)\bar{y^{2}}$$

$$=\left( n+1 \right)\beta^{2}-2n\bar{y_{b}}\beta+\left( n'+1 \right)\left( \alpha-E\left( \alpha| \beta,y \right) \right)^{2}+\left( n+n' \right)\bar{y^{2}}-\frac{\left( n'\bar{y_{a}}+\beta\right)^{2}}{n'+1}$$

$$=\left( \frac{n+n'+nn'}{n'+1} \right)\beta^{2}-2\left( n\bar{y_{b}}+\frac{n'}{n'+1}\bar{y_{a}} \right)\beta+\left( n'+1 \right)\left( \alpha-E\left( \alpha| \beta,y \right) \right)^{2}+\left( n+n' \right)\bar{y^{2}}-\frac{{n'}^{2}}{n'+1}\left( \bar{y_{a}} \right)^{2}$$

$$=\left( \frac{n+n'+nn'}{n'+1} \right)\left[ \beta^{2}-2\left( \frac{\left( n+nn' \right)\bar{y_{b}}+n'\bar{y_{a}}}{n+n'+nn'} \right)\beta\right]+\left( n'+1 \right)\left( \alpha-E\left( \alpha| \beta\right) \right)^{2}+\left( n+n' \right)\bar{y^{2}}-\frac{{n'}^{2}}{n'+1}\left( \bar{y_{a}} \right)^{2}$$

$$=\left( \frac{n+n'+nn'}{n'+1} \right)\left( \beta-\hat{\beta} \right)^{2}+\left( n'+1 \right)\left( \alpha-E\left( \alpha| \beta,y \right) \right)^{2}+\left( n+n' \right)\bar{y^{2}}-\frac{{n'}^{2}}{n'+1}\left( \bar{y_{a}} \right)^{2}-\frac{\left[ \left( n+nn' \right)\bar{y_{b}}+n'\bar{y_{a}} \right]^{2}}{\left( n+n'+nn' \right)\left( n'+1 \right)}$$

$$=\left( \frac{n+n'+nn'}{n'+1} \right)\left( \beta-\hat{\beta} \right)^{2}+\left( n'+1 \right)\left( \alpha-E\left( \alpha| \beta,y \right) \right)^{2}+\left( n+n' \right)\bar{y^{2}}-\frac{{n'}^{2}}{n'+1}\left( \bar{y_{a}} \right)^{2}-\frac{\left( n+nn' \right)^{2}\left( \bar{y_{b}} \right)^{2}+{n'}^{2}\left( \bar{y_{a}} \right)^{2}+2n'\left( n+nn' \right)\bar{y_{a}} \bar{y_{b}}}{\left( n+n'+nn' \right)\left( n'+1 \right)}$$

$$=\left( \frac{n+n'+nn'}{n'+1} \right)\left( \beta-\hat{\beta} \right)^{2}+\left( n'+1 \right)\left( \alpha-E\left( \alpha| \beta,y \right) \right)^{2}+\left( n+n' \right)\bar{y^{2}}-\frac{n^{2}\left( n'+1 \right)\left( \bar{y_{b}} \right)^{2}+{n'}^{2}\left( n+1 \right)\left( \bar{y_{a}} \right)^{2}-2nn'\bar{y_{a}} \bar{y_{b}}}{\left( n+n'+nn' \right)}$$

$$=\left( \frac{n+n'+nn'}{n'+1} \right)\left( \beta-\hat{\beta} \right)^{2}+\left( n'+1 \right)\left( \alpha-E\left( \alpha| \beta,y \right) \right)^{2}+\left( n+n' \right)\bar{y^{2}}-n'\left( \bar{y_{a}} \right)^{2}-n\left( \bar{y_{b}} \right)^{2}+\frac{nn'\left( \bar{y_{b}} \right)^{2}+nn'\left( \bar{y_{a}} \right)^{2}-2nn'\bar{y_{a}} \bar{y_{b}}}{\left( n+n'+nn' \right)}$$

$$=\left( \frac{n+n'+nn'}{m+1} \right)\left( \beta-\hat{\beta} \right)^{2}+\left( n'+1 \right)\left( \alpha-E\left( \alpha| \beta,y \right) \right)^{2}+{SSR}_{MLE}+\frac{nn'}{\left( n+n'+nn' \right)}\left( \bar{y_{a}}-\bar{y_{b}} \right)^{2}$$

$$=\left( \frac{n+n'+nn'}{n'+1} \right)\left( \beta-\hat{\beta} \right)^{2}+\left( n'+1 \right)\left( \alpha-E\left( \alpha| \beta,y \right) \right)^{2}+{SSR}_{1}.$$

The marginal probability of the data under *M*_1_ is

$$p\left( y | M_{1} \right)=\int_{0}^{\infty} \int_{-\infty}^{\infty} \int_{-\infty}^{\infty} p\left( {\hat{\alpha},\sigma}^{2} \right)p\left( \alpha\left| {\beta,\sigma}^{2} \right. \right)\prod_{i=j}^{n} p\left( y_{j}\left| \mu=\beta,\sigma^{2} \right. \right)\prod_{i=n+1}^{n+n'} p\left( y_{j}\left| \mu=\alpha,\sigma^{2} \right. \right)d\alpha d\beta d\sigma^{2},$$

$$p\left( y | M_{1} \right)=\left( 2\pi\right)^{-\frac{n+n'+1}{2}}\int_{0}^{\infty} \left( \frac{1}{\sigma^{2}} \right)^{\frac{\left( n+n'+1 \right)}{2}+1}\int_{-\infty}^{\infty} \int_{-\infty}^{\infty} \exp\left[ -\frac{1}{2\sigma^{2}}\left( \left( \alpha-\beta\right)^{2}+\sum_{j=1}^{n} \left( y_{j}-\beta\right)^{2}+\sum_{j=n+1}^{n+n'} \left( y_{j}-\alpha\right)^{2} \right) \right]d\alpha d\beta d\sigma^{2},$$

$$p\left( y | M_{1} \right)=\left( 2\pi\right)^{-\frac{n+n'+1}{2}}\int_{0}^{\infty} \left( \frac{1}{\sigma^{2}} \right)^{\frac{\left( n+n'+1 \right)}{2}+1}\exp\left( -\frac{{SSR}_{1}}{2\sigma^{2}} \right)\left( \int_{-\infty}^{\infty} \exp\left[ -\frac{n+n'+nn'}{2\sigma^{2}\left( m+1 \right)} \right]d\beta\right)$$

$$\times\left( \int_{-\infty}^{\infty} \exp\left[ -\frac{\left( m+1 \right)}{2\sigma^{2}}\left( \alpha-E\left( \alpha| \beta,y \right) \right)^{2} \right]d\alpha\right)d\sigma^{2},$$

$$p\left( y | M_{1} \right)=\left( 2\pi\right)^{-\frac{n+n'-1}{2}}\left( n+n'+nn' \right)^{-\frac{1}{2}}\int_{0}^{\infty} \left( \frac{1}{\sigma^{2}} \right)^{\frac{\left( n+n'-1 \right)}{2}+1}\exp\left( -\frac{{SSR}_{1}}{2\sigma^{2}} \right) d\sigma^{2},$$

$$p\left( y | M_{1} \right)=\left( 2\pi\right)^{-\frac{n+m-1}{2}}\left( n+n'+nn' \right)^{-\frac{1}{2}}\text{}\left( \frac{n+n'-1}{2} \right)\left( {SSR}_{1} \right)^{-\left( n+n'-1 \right)/2}.$$

The Bayes factor is

$$BF=\frac{p\left( y\left| M_{0} \right. \right)}{p\left( y\left| M_{1} \right. \right)}=\sqrt{\frac{n+n'+nn'}{n+n'}}\left( \frac{{SSR}_{1}}{\left( n+n' \right)\left( \bar{y^{2}}-\left( \bar{y} \right)^{2} \right)} \right)^{\frac{n+n'-1}{2}}. (S5)$$

Equations (S3), (S4) and (S5) together are equivalent to equations (10), (11), and (12) of the main paper.

## B.1. Derivation of posterior distribution for sampling variances

Under the null hypothesis with non-paired data, the data have the same mean, but the null hypothesis says nothing about sampling variances for the treatment and control data. In section A, the variances were treated as identical. Here we treat them as unrelated. Suppose that

$y_{j}=\beta+{\sigma\varepsilon}_{j}, j=1,\ldots, n$,

$y_{j}=\beta+{\sigma'\varepsilon}_{j}, j=n+1,\ldots, n+n'$.

where *ε_j_* are independent and identically Gaussian with mean zero and variance 1, *σ*^2^ is the variance of the control data, and *σ*’^2^ is the sampling variance of the treatment data.

To calculate the posterior predictive variance of a new treatment data point minus a new control data point, we need (up to proportionality) the posterior distribution of *σ*^2^ and *σ*’^2^, which we derive here.

Prior distribution

We set

$p\left( \beta,\sigma^{2},\sigma^{'2} \right)\propto\frac{1}{\sigma^{2}}\frac{1}{\sigma^{'2}}$.

Posterior distribution

Define

$$\bar{y_{a}}=\frac{1}{n'}\sum_{j=n+1}^{n+n'} y_{j},$$

$$\bar{y_{b}}=\frac{1}{n}\sum_{j=1}^{n} y_{j},$$

$$SSR'=\sum_{j=n+1}^{n+n'} \left( y_{j}-\bar{y_{a}} \right)^{2},$$

$$SSR=\sum_{j=1}^{n} \left( y_{j}-\bar{y_{b}} \right)^{2}.$$

Before dealing with the posterior distribution of *σ*^2^ and *σ*’^2^, we re-arrange the quadratic expression in *β* to ease the integration that will follow.

$$\frac{n\left( \beta-\bar{y_{b}} \right)^{2}}{\sigma^{2}}+\frac{n'\left( \beta-\bar{y_{a}} \right)^{2}}{{\sigma'}^{2}}$$

$$=\frac{n}{\sigma^{2}}\left( \beta^{2}-2\beta\bar{y_{b}}+{\bar{y_{b}}}^{2} \right)+\frac{n'}{{\sigma'}^{2}}\left( \beta^{2}-2\beta\bar{y_{a}}+{\bar{y_{a}}}^{2} \right),$$

$$=\left( \frac{n}{\sigma^{2}}+\frac{n'}{{\sigma'}^{2}} \right)\beta^{2}-2\beta\left( \frac{n\bar{y_{b}}}{\sigma^{2}}+\frac{n'\bar{y_{a}}}{{\sigma'}^{2}} \right)+\frac{n{\bar{y_{b}}}^{2}}{\sigma^{2}}+\frac{n'{\bar{y_{a}}}^{2}}{{\sigma'}^{2}},$$

$$=\left( \frac{n}{\sigma^{2}}+\frac{n'}{{\sigma'}^{2}} \right)\left[ \beta^{2}-2\beta\left( \frac{n{\sigma'}^{2}\bar{y_{b}}+n^{'}\sigma^{2}\bar{y_{a}}}{{n{\sigma'}^{2}+n}^{'}\sigma^{2}} \right) \right]+\frac{n{\bar{y_{b}}}^{2}}{\sigma^{2}}+\frac{n'{\bar{y_{a}}}^{2}}{{\sigma'}^{2}},$$

$$=\left( \frac{n}{\sigma^{2}}+\frac{n'}{{\sigma'}^{2}} \right)\left[ \beta-\left( \frac{n{\sigma'}^{2}\bar{y_{b}}+n^{'}\sigma^{2}\bar{y_{a}}}{{n{\sigma'}^{2}+n}^{'}\sigma^{2}} \right) \right]^{2}+\frac{n{\bar{y_{b}}}^{2}}{\sigma^{2}}+\frac{n'{\bar{y_{a}}}^{2}}{{\sigma'}^{2}}-\frac{\left( n{\sigma'}^{2}\bar{y_{b}}+n^{'}\sigma^{2}\bar{y_{a}} \right)^{2}}{{n{\sigma'}^{2}+n}^{'}\sigma^{2}}.$$

For the full set of parameters, the posterior distribution is,

$$p\left( \beta,\sigma^{2},\sigma^{'2}|y \right)\propto\left( \sigma^{2} \right)^{-\left( \frac{n}{2}+ 1 \right)}\left( \sigma^{'2} \right)^{-\left( \frac{n'}{2}+1 \right)}\exp\left[ -\frac{\sum_{j=1}^{n} \left( \beta-y_{j} \right)^{2}}{{2\sigma}^{2}}-\frac{\sum_{j=n+1}^{n'} \left( \beta-y_{j} \right)^{2}}{{2\sigma}^{2}} \right],$$

$$=\left( \sigma^{2} \right)^{-\left( \frac{n}{2}+ 1 \right)}\left( \sigma^{'2} \right)^{-\left( \frac{n'}{2}+1 \right)}\exp\left[ -\frac{1}{2}\left( \frac{n\left( \beta-\bar{y_{b}} \right)^{2}}{\sigma^{2}}+\frac{n'\left( \beta-\bar{y_{a}} \right)^{2}}{{\sigma'}^{2}}+\frac{SSR}{\sigma^{2}}+\frac{SSR'}{{\sigma'}^{2}} \right) \right],$$

$$=\left( \sigma^{2} \right)^{-\left( \frac{n}{2}+ 1 \right)}\left( \sigma^{'2} \right)^{-\left( \frac{n'}{2}+1 \right)}\exp\left[ -\frac{1}{2}\left\{ \left( \frac{n}{\sigma^{2}}+\frac{n'}{{\sigma'}^{2}} \right)\left[ \beta-\left( \frac{n{\sigma'}^{2}\bar{y_{b}}+n^{'}\sigma^{2}\bar{y_{a}}}{{n{\sigma'}^{2}+n}^{'}\sigma^{2}} \right) \right]^{2}+\frac{n{\bar{y_{b}}}^{2}}{\sigma^{2}}+\frac{n'{\bar{y_{a}}}^{2}}{{\sigma'}^{2}}-\frac{\left( n{\sigma'}^{2}\bar{y_{b}}+n^{'}\sigma^{2}\bar{y_{a}} \right)^{2}}{{n{\sigma'}^{2}+n}^{'}\sigma^{2}}+\frac{SSR}{\sigma^{2}}+\frac{SSR'}{{\sigma'}^{2}} \right\} \right].$$

Next we marginalize *β* to eliminate it from the posterior distribution.

$p\left( \sigma^{2},\sigma^{'2}|y \right)=\int_{-\infty}^{\infty} p\left( \beta,\sigma^{2},\sigma^{'2}|y \right)d\beta,$

$$\propto\left( \sigma^{2} \right)^{-\left( \frac{n}{2}+ 1 \right)}\left( \sigma^{'2} \right)^{-\left( \frac{n'}{2}+1 \right)}\exp\left[ -\frac{1}{2}\left\{ \frac{n{\bar{y_{b}}}^{2}}{\sigma^{2}}+\frac{n'{\bar{y_{a}}}^{2}}{{\sigma'}^{2}}-\frac{\left( n{\sigma'}^{2}\bar{y_{b}}+n^{'}\sigma^{2}\bar{y_{a}} \right)^{2}}{{n{\sigma'}^{2}+n}^{'}\sigma^{2}}+\frac{SSR}{\sigma^{2}}+\frac{SSR'}{{\sigma'}^{2}} \right\} \right]\int_{-\infty}^{\infty} \exp\left\{ -\frac{1}{2}\left( \frac{n}{\sigma^{2}}+\frac{n'}{{\sigma'}^{2}} \right)\left[ \beta-\left( \frac{n{\sigma'}^{2}\bar{y_{b}}+n^{'}\sigma^{2}\bar{y_{a}}}{{n{\sigma'}^{2}+n}^{'}\sigma^{2}} \right) \right]^{2} \right\}d\beta,$$

$$=\left( \sigma^{2} \right)^{-\left( \frac{n}{2}+ 1 \right)}\left( \sigma^{'2} \right)^{-\left( \frac{n'}{2}+1 \right)}\left( \frac{n}{\sigma^{2}}+\frac{n'}{{\sigma'}^{2}} \right)^{\frac{1}{2}}\exp\left[ -\frac{1}{2}\left( \frac{n{\bar{y_{b}}}^{2}}{\sigma^{2}}+\frac{n'{\bar{y_{a}}}^{2}}{{\sigma'}^{2}}-\frac{\left( n{\sigma'}^{2}\bar{y_{b}}+n^{'}\sigma^{2}\bar{y_{a}} \right)^{2}}{{n{\sigma'}^{2}+n}^{'}\sigma^{2}}+\frac{SSR}{\sigma^{2}}+\frac{SSR'}{{\sigma'}^{2}} \right) \right].$$

This is the final expression. It is not a standard distribution, but it is easy to generate Markov chain Monte Carlo samples from it.
